# Advanced vaccinology training globally: Update and impact of the COVID-19 crisis

**DOI:** 10.1016/j.vaccine.2022.08.029

**Published:** 2022-09-16

**Authors:** Carine Dochez, Philippe Duclos, Noni MacDonald, Christoph Steffen, Paul-Henri Lambert, the Global Vaccinology Training Collaborative

**Affiliations:** a University of Antwerp, Network for Education and Support in Immunisation, Universiteitsplein 1, 2610 Antwerp, Belgium; b University of Geneva, Centre for Vaccinology, 1 Rue Michel Servet, 1211 Geneva 4, Switzerland; c Department of Pediatrics, Dalhousie University, IWK Health Centre, 5850/5980 University Ave, Halifax, Nova Scotia B3K 6R8, Canada; d World Health Organization, Geneva, Switzerland

**Keywords:** Vaccines, Vaccinology, Education, Courses, Global

## Abstract

The rapid development of innovations and new technologies, the focus on the life-course approach to immunization and equity, and the prevalent hesitancy towards vaccines requires immunization staff to be well-trained and updated regularly in order to deliver quality immunization services to the public. The need for advanced vaccinology training is therefore paramount. In preparation for a second Global Workshop on Advanced Vaccinology Training that took place in March 2022, this paper presents the results of a survey aiming to provide a thorough update of a landscape analysis on advanced vaccinology courses conducted in 2018 and a look at the impact of the COVID-19 crisis. Thirty-three course organizers responded to a survey to provide information on their respective course. Of those, 17 courses are short courses, 11 post-graduate courses and 5 are Master level courses. Most courses are organized on an annual basis. Even though some courses were not sustained overtime, the number of courses has been increasing during the last few years, and at least one vaccinology course is now being offered in each WHO region. Although the training capacity has increased tremendously, the need still exceeds the capacity and many courses have way more applicants than they can select. The most frequent challenges reported included sustainable funding and identifying faculty. The COVID-19 pandemic impacted the delivery of several vaccinology courses, which have been postponed or reformatted to an online or hybrid training event. An e-portal of the global collaboration has been established to facilitate communication between the different courses and to assist future course participants to identify the most suitable course for their needs.

## Highlights

• A total of 33 advanced vaccinology courses were in existence in 2021.
• Some vaccinology courses were not sustained since 2018.
• The number of vaccinology courses has been increasing during the last few years, with courses offered in each WHO region.
• The COVID-19 pandemic resulted in the cancellation or postponement of some vaccinology courses.
• Due to the COVID-19 pandemic, an increased number of courses are using an online or hybrid format.

## 1 Introduction

The ambitious goals set by the Immunization Agenda 2030 (IA2030) for the decade 2021–2030 builds on the lessons learnt from the Global Vaccine Action Plan (GVAP), taking into account ongoing and new challenges posed by infectious diseases [1], [2], [3]. To develop effective and safe vaccines of good quality, and to deliver high-quality immunization services to the public, a well-trained, skilled, knowledgeable, and motivated workforce with good communication skills is needed. The IA2030 clearly outlines the need for an adequate and effective workforce, as well as the need to strengthen evidence-based decision-making [3].

The rapid development of innovations and new technologies, the focus on the life-course approach to immunization and on equitable access and use of vaccines [3], requires immunization staff to be updated regularly if they have to cope with strategic changes and technical advancements. While life-course immunization has already received increased attention in the past years, the COVID-19 pandemic really emphasized the potential importance of vaccinating beyond infancy, childhood and adolescents and reaching out to other age groups as well, including adults, elderly and pregnant women depending on the vaccine-preventable diseases and their specificities.

Already in 1999, a group of vaccinology experts recognized the need for advanced vaccinology education to strengthen the immunization workforce around the world, and the first Advanced Vaccinology Course (ADVAC) took place in 2000 [4]. The general objective of a vaccinology course is ‘’to master the basic principles and specificities of a vaccine, how it is developed, produced and utilized, including its use within the immunization programme’’. Since 2000, several advanced vaccinology courses in different regions of the world have been organized by a variety of partners, aiming to strengthen the vaccinology education for researchers, clinicians, educators, regulators, manufacturers and public health officials. Especially the last decade has seen a growth in the number of vaccinology courses [5], [6], [7], [8].

A first Global Vaccinology Training workshop was organized in November 2018, bringing together leaders from vaccinology courses around the world to carry out an extensive review of the existing courses worldwide, in order to identify education gaps and future needs and discuss potential collaboration [4]. In preparation for this first workshop, directors of 26 of 39 identified advanced vaccinology courses who participated in this first workshop completed a questionnaire to gather information on the respective courses, including but not limited to educational methods, audience, funding, and challenges. The results of this landscape analysis were published in 2020 [9].

Following this first workshop an informal collaboration among the different courses was set up with regular 6-monthly tele-conferences between members of the collaboration and a number of workstreams. An e-portal was established to help those seeking more advanced training in vaccinology identify vaccinology courses which could be of interest to them and to facilitate exchange between the members of the collaboration. After about three years of informal functioning of the collaboration, a second global workshop in 2022 was planned to further the aims of the global collaboration on advanced vaccinology training, assist courses in addressing challenges in priority areas and facilitate interactions and exchange of information [10]. In preparation for this second workshop an attempt was made to update the landscape analysis conducted in 2018 in a comprehensive manner and see how the courses scenery had evolved, particularly in the context of the challenges posed by the protracted COVID-19 crisis. This paper aims to describe the updated landscape analysis of advanced vaccinology courses.

## 2 Methods

This landscape analysis was designed as a cross-sectional survey of existing specific advanced vaccinology courses around the world, including short courses, and Master programs and other non-Master post-graduate courses and following the same inclusion criteria as that conducted in 2018 [9].

We endeavored to contact all courses pre-identified in 2018, as well as the courses who had since joined the collaboration and conducted an exhaustive search for additional courses on the web using vaccinology training keywords and via publications. The first two authors, CD and PD, independently attempted this search. We also attempted to contact courses listed in publications and contacted various professional networks asking for the identification of vaccinology courses at national level. This included the Global NITAG Network and partners from the Network for Education and Support in Immunisation. Course Directors and various experts were also asked to contribute to the identification of additional courses.

University-based undergraduate (i.e. pre-service) courses that only focused on one specific issue/area of vaccinology, and periodic courses or symposia on vaccinology were excluded as well as graduate courses and or vaccinology modules being offered as part of a wider scope degree e.g. a course on infectious diseases. Also post-doctoral research training programs were excluded. The list of courses identified was shared with immunization officers serving as focal points for training and NITAGs in the 6 WHO regional offices to ensure that no known courses would have been missed.

The course directors of the identified courses were asked to complete a standardized questionnaire, which was based on the questionnaire from 2018, including additional questions related specifically to any recent change or challenge which would have occurred since 2018. The questionnaire comprised of 33 questions including: information on the course organization, coordination, collaborating partners and funding sources, course participant selection process including background, geographic and their economic status; course objectives and curricula, format of training, evaluation process, post-course activities and communication, and questions on the impact of the COVID-19 pandemic. The questionnaire was sent by e-mail to the identified course directors, allowing initially for a 3-month period for completion. Email reminders and follow-up phone calls were made to ensure completion of the questionnaires and clarify answers as necessary.

For courses potentially identified from various sources but from which no answer could be elicited after many reminders and attempts to contact the presumed organizers directly and indirectly and for which no evidence of recent orchestration of the courses could be found, it was assumed that these courses were no longer in existence.

Data were subsequently entered in Microsoft® Excel 2018 (version 16.16.4, Redmond, WA, USA). We summarized data descriptively using frequencies for categorical variables and measures of central tendency for continuous variables.

The survey was sent only to the course directors only. No personal or sensitive information nor patient data were collected and no vulnerable groups were included. Therefore, it did not meet the criteria for submission for institutional Ethics Board review.

## 3 Results

A total of 33 courses were identified that fit the criteria stated above. This included 23 of the courses previously identified and included in the previous landscape analysis and an additional 10 courses. These additional courses included some courses newly established/being established as well as additional courses which existed previously but were not identified in the previous search for courses. Of the courses which were included in the previous landscape analysis, 3 of them had been discontinued for a number of reasons including lack of funding or lack of champion to ensure continuity of the course or change in priorities; while one is on hold until new funding has been identified.

Of the 33 courses contacted, 30 (90.9 %) completed the new questionnaire. Three courses (Pasteur international Course, MOOC Pasteur and the Epidemiological evaluation of vaccines: efficacy, safety and policy organized by LSHTM) did not complete the new questionnaire due to lack of time, but indicated that the data provided in 2018 were still valid and that nothing much had changed, and as such were included in the overview (data summarized in Table 1). For these courses that did not return the updated questionnaire, information provided in the previous questionnaire from 2018 was used and the cumulative number of trainees in the Table was not updated.

**Table 1:** Characteristics of selected Advanced Vaccinology Courses around the world by 2021.

Name	WHO Region	Country	Year Established	Reach	Language	Type of course	Course delivery before COVID-19 pandemic	Frequency of course	Total Participants Trained	Number participants/course	Type of participants*
VACFA Annual African Vaccinology Course	AFR	South Africa	2004	R	E	Short	Face-to-face	Annual	1000	75	Public health (60 %), academics (15 %), industry (10 %), students (10 %) and regulatory authorities (5 %)
ALIVE Masters in vaccinology		South Africa	2019	R	E	MSc	Face-to-face	Annual	24	10–15	Medical and allied medical practitioners and scientist, public health scientists
ALIVE African Advanced Vaccinology Course		South Africa	2016	G	E	Short	Face-to-face	Every 2 years	100	50–55	Majority academia and public health; few industry
SAVIC Higher Certificate in Vaccinology		South Africa	2019	N	E	Post grad	Online	Annual	36	27	Public health (74 %), regulatory authorities (23 %), academics (3 %)
SAVIC Vaccinology Short Course		South Africa	2016	N	E	Short	Face-to-face	Annual	151	16–20	Public health (46 %), academics (31 %), regulatory authorities (18 %), industry (5 %)
ECAVI Vaccinology Course for Health Professionals		Uganda	2016	R	E	Short	Face-to-face	Annual	650	75–100	Academia, public health, industry, research scientists
Master of Science in Vaccinology		Zimbabwe	2021	N	E	MSc	Blended	Every 2 years	7	10–20	Students not yet in job setting
Vaccinology in Africa Master’s Level Course		Rotating	2013	R	E	Short	Face-to-face	Every 2 years	183	30–41	Academics (90 %), public health (10 %)
International Course on Vaccinology		Senegal/Burkina Faso/Morocco	2012	SR	F	Post grad	Online	Annual	275	30–40	Most are public or private professionals
Ciro de Quadros Vaccinology Course for Immunisation Managers	AMR	Argentina	2011	R	S	Short	Face-to-face	Annual/Every 2 years	281	30–40	Public health, academics, government
Vaccinology & Immunotherapeutics graduate program		Canada	2008	G	E	Post grad	Face-to-face	Annual/Every 2 years	117	9–10	Graduate students enrolled in MSc and PhD degree programs
International Advanced Vaccinology Course		Chile	2018	R	S	Post grad	Face-to-face	Annual	106	30–56	Industry, regulatory authorities, national immunization program, academics, Fellows (ID, immunology, paediatrics); PhD students
Latin America Online Vaccinology Course		Mexico	2009	R	S	Post grad	Online	Annual	7200	30–500	Public health, regulatory authorities, academics, students, industry
Clinical Vaccinology Course		USA	2006	N	E	Short	Face-to-face	Annual	4500	250	Mostly public health and primary care
NITAG Vaccinology Course	EMR	Jordan	2021	R	E/F	Short	Face-to-face	Planned annual	NA	25	NITAG chairs and members, NITAG secretariat
Inter-University Diploma in Vaccinology		Morocco	2005	G	F	Post grad	Online	Every 2 years	NR	35–40	Health professionals
Summer course on Vaccinology for students	EUR	Belgium	2009	R	E	Short	Face-to-face	Annual	430	50	Majority students, few industry and public health
Certificat Interuniversitaire en Vaccinologie		Belgium	2021	R	F	Post grad	Blended	Annual	NA	50	Health professionals, public health institutions, industry, students, international organizations
ADVAC Advanced Course of Vaccinology		France	2000	G	E	Short	Face-to-face	Annual	1284	75	Public health (51 %), industry (25 %), academics (17 %), regulatory authorities (1.5 %), international organizations (5.5 %)
Cours International Francophone de Vaccinologie		France	2006	G	F	Short	Face-to-face	Every 2 years	220	15–25	Academia, industry, public health, regulatory authorities
MOOCS Vaccinology**		France	2015	G	E	Post grad	Online	Annual	5500	2000–3000	Academia, industry, public health, regulatory authorities, students
Institut Pasteur International Vaccinology Course**		France	2008	G	E	Post grad	Face-to-face	Annual	274	28	Academics (55 %), public health (35 %), regulatory authorities (5) and industry (5)
Leading International Vaccinology Education (LIVE)		France/Belgium/Spain	2015	G	E	MSc	Face-to-face	Annual	125	25	Mainly academics
Master in Vaccinology and Drug Development		Italy	2009	N	E	MSc	Face-to-face	Annual	104	20	Majority supranational organizations, followed by Clin dev, MoH and academics
Vaccines and Vaccination Strategies		Italy	2001	N	I	Post grad	Face-to-face	Annual	650	35–50	Half medical specialization students, half public health
Development and Application of Vaccines in Global Health		Spain	2014	G	E	Post grad	Face-to-face	Annual	114	10–24	Education, international organizations, MSc/PhD students, NGOs, private sector, health sector, researchers
IMVACC International Master of Vaccinology		Switzerland	2016	G	E	MSc	Online	Annual	34	5–15	Majority academia, followed by industry, public health and regulatory authority
Epidemiological evaluation of vaccines: efficacy, safety and policy**		UK	2009	G	E	Short	Face-to-face	Annual	164	30–40	Public health (60 %), academics (15 %), industry (10 %), regulatory (5 %) and others (10 %)
Oxford vaccinology course		UK	2009	G	E	Short	Face-to-face	Annual	212	18–24	Academia (60 %), industry (20 %), regulatory authorities (20 %)
Advanced Vaccinology Course in India (INDVAC)	SEAR	India	2010	G	E	Short	Face-to-face	Annual	378	30–50	Academics (67 %), industry (15 %), public health (11 %), regulatory authorities (7 %)
Advanced vaccinology for the Asia-Pacific	WPR	Australia	2021/2022	R	E	Short	Face-to-face	NR	NA	50	NITAGs, regulatory, MoH, research scientists, clinicians, others
Chinese Vaccinology Course (CNVAC)		China	2018	N	C/E	Short	Face-to-face	Annual	155	80	Public health (41 %); industry (30 %), academics (20 %), regulatory (7 %), NGOs (2 %)
IVI International Vaccinology Course		Republic of Korea	2000	G	E	Short	Face-to-face	Annual	1600	207	Government (40 %), academia (22 %), private companies (18 %), NGOs (13 %), Hospitals (7 %)

Notations: Regions: AMR: Americas, AFR: Africa, EUR: Europe, WPR: Western Pacific, SEAR: South East Asia; Reach: N: National, SR: sub regional, R: Regional, G: Global; Language: C: Chinese, E: English, F: French, S: Spanish; NR: Not reported; NA: Not applicable.

* Not all courses provided quantified information about the distribution of participants by professional categories.

** For these courses 2018 data was used and the cumulative number of trainees was not updated.

Of the 33 advanced vaccinology courses, 16 (48.5 %) reported targeting global, 11 (33.3 %) regional and sub-regional, and 6 (18.2 %) national audiences. Most courses are organized on an annual basis 24 (72.7 %), while the remaining courses are offered every other year or with a lower frequency. It is to be noted that the COVID-19 crisis seriously impacted vaccinology courses. Some had to be delayed or cancelled in 2020 or 2021 (see below section on the impact of the COVID-19 crisis for more details).

Of the courses offered, 17 are short courses, 11 are non-Master post-graduate courses and 5 are Master level courses. The duration of the short courses varies from 2 to 11 teaching days. Master programs have a duration of 2 years, except the Master course in Siena which has a duration of 12 months, a reduction from the previous 18 months.

Since the delivery of the first advanced vaccinology courses in 2000, the number of courses has continued to expand globally. While the first courses were located in Europe (France and Germany) and in the Western Pacific Region (South Korea), courses are now established in all WHO regions, with the majority of the courses being delivered in the European and African region (Table 1). Courses targeting a regional and global audience tend to have larger student participation as compared to national courses, except for the National Foundation for Infectious Diseases (NFID) course in the United States which has quite a large number of participants (Table 1).

In line with the significant increase in the number of courses, the number of professionals who have been trained overall also has dramatically increased. Courses located in the European and the Americas regions have had the largest number of trainees (n = 9037 (including 5534 online participants) and n = 12204 (including 7306 online participants) respectively) followed by those in the African region (n = 2419 (including 311 online participants); while those located in Western Pacific (n = 1755) and South East Asia (n = 387) regions trained the least number of participants. An advanced vaccinology course was just established in the Eastern Mediterranean region and the first edition of the course was delayed because of the COVID-19 crisis.

### 3.1 Participants’ selection

Most of the courses (n = 30, 91 %) have a process in place to select their participants by taking into account their educational background, vaccinology experience, motivation and potential impact of course attendees at institutional and national level. Of those, 3 courses implement a first come – first serve criterion when the other requirements have been met, while 3 other courses are working with a nomination process mainly through the *EPI* program. Two master programs conduct interviews as part of their selection process. Four courses specifically stated paying attention to the geographical distribution of participants.

Only 3 courses did not have a selection process in place and welcomed any interested participant. The different types of participants are summarized in Table 1. The majority of courses select participants who are working in public health (for the courses who reported percentages of participants, range 10–74 %) or academia (range 3–90 %). Most course also have attendance of participants from industry or regulatory authorities, but the numbers are much lower as compared to public health and academia (vaccine industry (range 5–30 %) and regulatory authorities (range 1.5–23 %)). While most participants are mid-career level, the entire career range is seen in many of these courses.

Some courses also include participants from international organization and Non-Governmental Organization (NGOs). One newly established course in EMR specifically focusses on NITAG chair and members and the NITAG secretariat. Three courses indicated to have a majority of students not yet in job attending their course, of which two are Master programs and one a summer course.

### 3.2 Courses’ structure

All courses use an educational syllabus that includes vaccine immunology, epidemiology, clinical trial design, immunization program strengthening, vaccine safety, ethics and topics on new vaccines (malaria, dengue, cholera, Ebola, COVID-19, etc.). All courses deliver most of this content via interactive lectures by international and national experts in vaccinology. Most (n = 27, 82 %) of the courses include small group exercises, case studies or special workshops as part of the teaching activities. Four courses also give the opportunity for participants to give a presentation. Four courses include site visits. Five courses indicated pre-reading requirements.

The majority of courses are delivered in English (n = 24, 73 %), followed by French (n = 4), Spanish (n = 3), Italian (n = 1) and Chinese (n = 1).

### 3.3 Evaluation and impact of the vaccinology courses

A total of 24 of the 33 courses reported their respective course evaluation system, of which 9 indicated using an online evaluation system. Seven courses reported to only track the participants’ attendance rate and evaluation of the participants through pre- and post-tests. Two courses stated they did not have a course evaluation in place. One course had external evaluators present throughout the course who reported their findings to the funding agencies.

All courses with a course evaluation system in place asked the participants to evaluate the scientific content of the course, as well as the administrative/logistics part. Participants evaluated each session, including content, quality of presentations and background materials, interaction of facilitators with participants, as well as the organization of the course, venue and time management. Most courses conduct this evaluation on a daily basis or after each session. Several courses also report having an overall evaluation at the end of the course, including the possibility of making suggestions, which are taken into consideration to make recommendations for next years’ course.

Measuring the long-term impact of the vaccinology courses on the career of participants is work in progress. One course reported having started the evaluation of course impact in 2019 and further expanded it to a full pre-course evaluation focused on assessment of needs and self-evaluation of knowledge and skills, which is repeated at the end of the course to assess impact. One of the newly established courses reported that a detailed impact analysis will be conducted; participants will be asked to provide feedback using an online tool prior to attending the course, immediate post-course and 6-months post-course to ascertain how knowledge gained during the course has translated into practice. Another course reported to be in the process of developing impact evaluation studies.

However, informal communication and contact through the different course alumni platforms, suggests that many vaccinology course alumni are active in governmental or non-governmental organizations at the global, regional or national level, or in academia or in the vaccine industry and that the courses helped them with their job and progress in their career, i.e. have remained active in vaccinology area post training.

### 3.4 Post-course activities

Eighteen of the courses reported having an active alumni network with periodic or regular contact with previous participants, while 5 courses reported informal networking activities with their alumni. Eight courses spontaneously mentioned to give access to teaching and reading materials to alumni, while four courses reported to organize regular online webinars. One course mentioned to conduct supportive supervisory visits of their alumni. In addition, 10 courses reported supporting cascade training which is important for the development of new courses in different regions in the world, especially at the national and sub-national level by bringing vaccinology education closer to the areas of highest need.

### 3.5 Challenges

The most frequent challenges reported by courses were funding, faculty and curriculum (Table 2). The first and foremost concern was the sustainability of funding reported by 16 courses. Eight courses reported difficulties with the identification of suitable faculty, as many experts are already time constrained. Three courses noted the time needed for developing and updating the curriculum as challenging. Several courses were also challenged by the recruitment of the participants. Three courses mentioned specifically the variable educational background and lack of uniformity in work experiences of participants as a challenge to deliver the course with sufficient depth, identify common interest and manage expectations of all. One online course reported a high student drop-out rate. A number of courses also mentioned logistical issues in organizing their course, like administrative issues, finding suitable accommodation for participants and organizing the course from a distance (Table 2).

**Table 2:** Key challenges of Vaccinology Courses by type of course, 2021.

	Short course (n = 17)	Post graduate course (n = 11)	Master program (n = 5)	Total
Funding	11	1	4	16
Lecturers	3	4	1	8
Participants	3	3	0	6
Logistics	3	2	1	6
Teaching materials	0	2	1	3

### 3.6 Impact of COVID-19 pandemic

Of the 29 courses that were operational before the start of the COVID-19 pandemic, 23 courses used to be delivered face-to-face, while 6 courses were offered online (some courses with limited face-to-face activities like site visits). At the time of the survey 4 additional courses were under development for implementation in 2021/2022. This includes 2 face-to-face courses, 1 blended and 1 online course.

The negative impact of the COVID-19 pandemic on the courses included reduced funding; reduced number of students as face-to-face training is preferred; difficulties in finding sufficient lecturers as many of them are busy with the pandemic, reformatting the course to an online or hybrid event. Some courses, however, reported the benefits of moving to an online or hybrid course and reported a higher number of participants and the possibility to open it up to international students.

In 2020 only 3 courses were delivered as usual. This includes two online courses and one face-to-face national course held later during the year. Nine courses were postponed to 2021, 5 courses were delivered as hybrid courses, while 3 courses used a live-interactive event and 1 e-learning. The other courses did not respond or it was not applicable as the course was still under development. For the future, 22 courses mentioned they would consider virtual training, of which 4 already offered online training. Six of those courses would however prefer to return to face-to-face training, or at least to a form of blended training. Two courses clearly stated that they are not considering virtual training. The remaining courses did not respond to this question.

## 4 Discussion

Despite our efforts to have an exhaustive landscape analysis and to identify all existing specific advanced vaccinology courses around the world, we may have missed courses with no information easily accessible on the web nor published in indexed journals. Some may also argue that we applied our inclusion criteria too narrowly and that other university programs should have been surveyed. Despite this, we believe that the results are giving a fairly balanced picture of the current situation globally. It is clear that the situation has changed overtime and that the COVID-19 crisis and huge vaccine needs and related tremendous financial investments in vaccines/vaccinology may have resulted in new courses being established or soon to be established. The sustainability of some of the courses and/or new initiatives is challenging. This landscape analysis has noted a wealth of short-term advanced vaccinology training for health care workers, many university programs with a vaccine focus and or vaccine modules as part of wider scope courses, particularly in the USA as well as several additional very narrowly focused courses, such as training on safety or on regulatory aspects that were not the object of this analysis.

Since the landscape analysis from 2018, new courses emerged (Australia, Belgium, Chile, Jordan, South Africa (2 courses) and Zimbabwe), while several courses were discontinued. Three courses listed in the previous landscape analysis were not sustained, while one course is temporarily on hold until new funding has been identified. It is also to be noted that several of the courses identified in the previous attempt to identify courses but not included in the previous landscape analysis were not sustained and seem to have been more of one-time initiatives.

This review confirms the major conclusions from the 2018 landscape analysis and points to the further efforts globally implemented to meet the substantial demand for advanced vaccinology training with expansion of courses in all regions of the world and better access to trainees through language diversification of the courses. It is encouraging to see the beginning of the expansion of courses in the Eastern Mediterranean Region and the Western Pacific Region whereas the Eastern European area of Europe still remains poorly served. The language of the courses still represents a huge limitation for potential participants from some countries/regions. However, adding simultaneous interpretation to some of the existing course would limit smooth content delivery and decrease interaction between the participants and with the faculty. Efforts should be made to organize courses even in off years in languages such as Russian. This could help meet the demand.

In comparison with results reported previously [9], an additional 6496 advanced vaccinologists have been trained through specific advanced vaccinology courses and this is an under-estimate as we failed to secure an update for three of the courses. This still represents a small fraction of the pool of experts who need and are seeking training as demonstrated by the substantially larger number of applicants for courses each year. This growing need is in part fueled by the increasing speed with which people change jobs and change responsibilities nowadays as well as growth in the perception of the value of vaccines on health and wellbeing that COVID-19 has brought.

This updated landscape analysis highlights the similarities and differences in what, where, when, how, and for whom these advanced courses are offered. Further tracking of key indicators and future review of their content and breath of training will be relevant, as well as a reassessment of the range of participants trained to see if gaps have been filled.

The Bill & Melinda Gates Foundation, along with many of the host academic institutions and other national foundations have been at the forefront of providing the funding for this needed upsurge in advanced vaccinology training of the workforce. Despite the important contribution of the courses, funding sustainability continues to be perceived as one of the major threats to sustainability by vaccinology course directors and Governments only had a very limited financial contribution.

One of the concerns of a few courses is the diversity of participants regarding educational background and experience. One the one hand, this diversity is a great advantage, allowing participants to understand diverse perspective and to foster cross sectoral collaborations, but on the other hand, this has made it challenging to adapt the programs to sufficiently meet all needs and be understandable by all participants. This was only observed for the short courses and the non-Master post-graduate courses, not for the Master programs. This difference might be explained by noting that enrollment for a Master program has clear educational prerequisites.

The e-portal (icavt.org) of the International Collaboration on Advanced Vaccinology Training, whose primary aim is to facilitate identification of suitable training by those looking for such courses will hopefully play a role both in making the courses more visible and facilitating their identification by potential participants in need of advanced vaccinology training and in facilitation of selection of the most appropriate course to fit their need. This platform will also help grow and strengthen a workforce network to advance vaccinology around the globe. It will be most helpful if all advanced vaccinology courses registered on the platform. This has still not been achieved even with the courses identified and in spite of all these courses being approached and invited to register to have their course listed on the portal We certainly hope that new courses and courses which may have been missed will be interested to join the collaboration and be listed on the e-portal and that they will approach the collaboration’s secretariat (https://www.icavt.org/fr/contact).

The challenge of finding qualified lecturers was mainly a problem for both short and post-graduate courses. Only one Master program that is recently established mentioned this as a challenge. In principle Master programmers will be delivered when sufficient lecturers at the hosting university/college are identified.

Although as previously stated it is difficult to assess the overall contribution of the courses to the development of the immunization workforce across many disciplines, it is clear that these courses have enhanced the capacity of the trainees and allowed them to proceed in their vaccinology career in the various fields. According to the framework of Moore et al. [11], most of the courses measure the participation and satisfaction level of their participants, as well as evaluate their learning by pre- and post-tests. However, many alumni anecdotally have gone on to grow in their vaccinology responsibilities and contributions and have taken positions of vaccinology leadership at regional and global public health institutions and industry. Without a systematic impact analysis, is not clear what aspects of these courses have proven to be most useful and impactful. As a result, a major focus of discussions at the second global workshop on advanced vaccinology training was the assessment of needs, impact evaluation and how to make sure that the courses are sufficiently enabling their target audiences [10]. Evaluations being a weak point of many courses, we strongly recommend all courses to strengthen these evaluations and to particularly endeavor to look at the actual impact of the training on the participants’ practice.

Most participants from the advanced vaccinology courses, are professionals in their mid-career, therefore, it is not surprising that short intense courses continue to be the most frequent educational format used to deliver vaccinology training and foster networking between participants. New approaches to teaching allowing to reach more participants have been pushed by the COVID-19 crisis that may continue for some time. The COVID-19 crisis has also exemplified the need for adjusting the content of the courses quickly to new priorities/issues.

The fast-evolving vaccinology field and priorities and related need for updates calls for the establishment of updating opportunities/courses and this could more efficiently be mutualized between courses.

The proposed formalization of the International Collaboration on Advanced training would facilitate the organization of updates, as well as the sharing of some resources and experiences and solutions in dealing with challenges [10].

## Declaration of Competing Interest

The authors declare that they have no known competing financial interests or personal relationships that could have appeared to influence the work reported in this paper.

## Data Availability

Data will be made available on request.

## Appendix B Courses participating in the Global Vaccinology training collaborative

Advanced Course of Vaccinology (ADVAC), Mérieux Foundation and the University of Geneva;

Advanced Vaccinology for the Asia-Pacific;

Advanced Vaccinology Course in India (INDVAC), Christian Medical College, Vellore;

African Leadership in Vaccinology Expertise (ALIVE), Master of Science (MSc) (Med) in the field of Vaccinology, The University of the Witwatersrand, Johannesburg;

Afro-ADVAC-African Advanced Vaccinology course, African Leadership in Vaccinology Expertise (ALIVE), The University of the Witwatersrand, Johannesburg;

Annual African Vaccinology Course (AAVC), University of Cape Town;

Certificat Interuniversitaire en Vaccinologie, Université Libre de Bruxelles, Université Catholique de Louvain, Université de Liège, Belgium:

Chinese Vaccinology Course (CNVAC), Medical School, University of Chinese Academy of Sciences;

Ciro de Quadros Vaccinology Course for Immunization Managers in Latin America, Sabin Vaccine Institute;

Clinical Vaccinology Course, National Foundation for Infectious Diseases (NFID);

Cours international francophone de vaccinologie (CIFV), Université de Bordeaux - Service de santé des armées - Groupe d’études en préventologie;

Development and Application of Vaccines in Global Health, IS Global-Barcelona Institute for Global Health, University of Barcelona, Spain;

Diplôme International Inter Universitaire de Vaccinologie (DIUI), University of Dakar;

Diplôme International de Vaccinology, Cadi Ayyad University Marrakech;

Summer course on vaccinology for students, Antwerp University;

Vaccinology Course for Health Professionals, East Africa Centre for Vaccines and Immunization (ECAVI);

Epidemiological Evaluation of Vaccines: Efficacy, Safety and Policy, London School of Hygiene & Tropical Medicine;

Higher Certificate in Vaccinology (HCert), South African Vaccination and Immunisation Centre (SAVIC), Sefako Makgatho Health Sciences University;

International Advanced Vaccines Course, University of Chile;

International Vaccinology Course, Institut Pasteur Paris;

International Vaccinology Course, International Vaccine Institute (IVI), Seoul;

Latin American Online Vaccinology Course (DILVAC), Carlos Slim Foundation;

Leading International Vaccinology Education (LIVE), Master of Science;

Master of Advanced Studies of Vaccinology (IMVACC), University of Lausanne;

Master of Science in Vaccinology; University of Zimbabwe;

Master in Vaccinology and Pharmaceutical Clinical Development, University of Siena, Institute for Global Health;

MOOCS vaccinology, Institut Pasteur, Paris;

NITAG Vaccinology Course EMRO, GHD/EMPHNET and NESI/University of Antwerp;

Vaccinology in Africa MSc Level Course, Jenner Institute, University of Oxford;

University of Oxford Vaccinology programme (Human and Veterinary Vaccinology, Clinical Development and Biomanufacturing), Jenner Institute and University of Oxford;

Vaccines and Vaccination Strategies, University of FIorence, Italy;

Vaccinology and Immunotherapeutics, University of Saskatchewan;

Vaccinology Short Course (VSC). South African Vaccination and Immunisation Centre (SAVIC), Sefako Makgatho Health Sciences University.
